# Moving From Keywords to Contextual Meaning: A Commentary on Hybrid Bibliometric Synthesis in Health Research

**DOI:** 10.2196/102159

**Published:** 2026-06-03

**Authors:** Dimitrios Zikos

**Affiliations:** 1 Department of Healthcare Management and Leadership, College of Health Professions, Texas Tech University Health Sciences Center, 3601 4th Street, Lubbock, TX, 79430, United States, 1 9894301787

**Keywords:** bibliometrics, machine learning, semantic mapping, public health surveillance, social media

## Abstract

The fast growth of social media mining in health research has contributed to an invaluable but quite fragmented body of literature. As the amount of unstructured patient-reported data grows, traditional bibliometric analyses face methodological limitations, particularly regarding synonym fragmentation and arbitrary parameter selection. In their recent publication, “Thematic Mapping and Evolution of Social Media Mining in Health Research: Hybrid Bibliometric Synthesis,” Yang and Bohnet-Joschko attempt to address these flaws by introducing a semantic-structural (hybrid) bibliometric framework. This commentary evaluates the methodological innovations of their study and its departure from traditional syntactic keyword-matching tools. By combining citation-informed transformers (SPECTER2) and biomedical language models (PubMedBERT) and dimensionality reduction and density-based clustering, the authors created a reproducible pipeline. In their architecture, they start with foundational machine learning (statistical validity) before transitioning into large language models for qualitative synthesis. I will attempt to explain how this transition from syntactic mapping to semantic vector representation solves known challenges in evidence synthesis, naturally grouping conceptual synonyms without artificially forcing boundaries on the literature. Furthermore, I examine the practical implications of their temporal findings. Such real-time social media mining applications can be very useful for retrospective reporting and evaluating targeted public health interventions. While this pipeline offers high generalizability across disciplines, it also introduces a computational literacy barrier to some, and this re-emphasizes the need for data literacy for health professions. Ultimately, the study provides a transparent approach to informatics because mathematically validated frameworks are foundational for the future of evidence-driven public health policy and clinical decision-making.

## Introduction

The fast growth of social media mining (SMM) in health research has provided new opportunities to study public health. However, the volume of this literature has created a fragmented knowledge base that resists traditional evidence synthesis. In their recent article, “Thematic Mapping and Evolution of Social Media Mining in Health Research: Hybrid Bibliometric Synthesis,” Yang and Bohnet-Joschko [1] go beyond descriptive field mapping and introduce a more semantically focused framework for evidence synthesis by extending traditional bibliometric methods with a hybrid semantic-structural pipeline. Since the health care data resources and knowledge base have already become massive and complex, synthesizing literature needs to go beyond just “cataloging” publications. This commentary explores how their methodology attempts to overcome statistical limitations, optimizes analytical architecture, and supports contextually relevant public health applications.

## The Limitations of Traditional Keyword Matching

For over a decade, bibliometric reviews have mostly relied on out-of-the-box platforms like VOSviewer, but in some ways, those may reduce transparency regarding parameter sensitivity and clustering behavior [2]. They are created around prebuilt clustering algorithms (such as the Louvain method) that do not provide the researcher control over cluster thresholding, network normalizations, and parameter sensitivity. Additionally, these traditional methods are constrained by syntactic mapping: they treat keywords, titles, and abstracts of articles as isolated strings of text. For example, traditional tools group papers by vocabulary (linking a Twitter flu paper with a bird flu paper), while the hybrid model groups them by scientific intent. Unless a researcher manually creates a detailed thesaurus, the algorithm fragments the literature based on vocabulary rather than meaning. Similarly, legacy topic modeling methods like latent Dirichlet allocation (LDA) rely on a rigid “bag-of-words” assumption that ignores contextual syntax and forces researchers to predefine the number of topics, artificially “bounding” the data [3].

Yang and Bohnet-Joschko [1] attempted to solve this by moving from syntactic to semantic mapping (Table 1). By choosing to use SPECTER2 and PubMedBERT embeddings, they ingest the entire context of a text and convert documents into high-dimensional vectors [4,5]. Because these models understand semantic meaning, they group conceptual synonyms together, making fewer errors than the traditional string-matching tools. Furthermore, because SPECTER2 is pretrained on very large amounts of citation data, it makes the approach an interesting hybrid of text mining and citation analysis.

**Table 1.** Comparison of traditional bibliometric methods to Yang and Bohnet-Joschko’s [1] pipeline.

Dimension	Traditional bibliometric methods	Yang and Bohnet-Joschko’s [1] hybrid pipeline
Data processing	Syntactic mapping of isolated text strings	Semantic vector representation using context
Core algorithms	Out-of-the-box platforms (eg, VOSviewer) using Louvain or latent Dirichlet allocation	Citation-informed transformers (SPECTER2), biomedical models (PubMedBERT), UMAP^a^, and HDBSCAN^b^
Clustering	Relies on forced boundaries, often requiring predefined topic counts	Density-based clustering that mathematically isolates unassigned noise
Vocabulary handling	Fragments literature based on terminology unless a manual thesaurus is built	Naturally groups conceptual synonyms together based on scientific intent

a UMAP: uniform manifold approximation and projection.

b HDBSCAN: hierarchical density-based spatial clustering of applications with noise.

## Optimization of the Architecture

The strength of Yang and Bohnet-Joschko’s [1] methodology is in the sequencing of its analytical architecture. In the current era of artificial intelligence, it is tempting to feed raw data directly into a large language model. The study argues for an emerging informatics principle: structurally validated machine learning pipelines should precede large language model–assisted interpretation.

Yang and Bohnet-Joschko [1] validate their data structure using uniform manifold approximation and projection (UMAP) for dimensionality reduction [6] and hierarchical density-based spatial clustering of applications with noise (HDBSCAN) for structural clustering [7]. Unlike LDA, HDBSCAN does not require a predefined cluster count. It identifies naturally occurring dense regions of literature and mathematically isolates outlier papers rather than forcing them into irrelevant groups. This way, the subsequent thematic synthesis is grounded in reproducible data science.

## From Methodology to Public Health Practice

Beyond the methodological advantages, the study’s [1] temporal slicing is, for SMM, an example of moving from computational experimentation to real-world public health engagement. The prominence of application-driven clusters, such as infodemiology and sociopsychological determinants, shows well how SMM can be a useful tool for community health surveillance [8]. Accurate SMM is essential for evaluating localized, real-world health initiatives. For instance, evaluating colorectal cancer screening prevention campaigns in Texas requires an understanding of localized, real-time community sentiment, barriers to access, and sociopsychological hesitation. SMM captures this narrative, augmenting structured retrospective federal tools that often lag by months or years. Such real-time sentiment surveillance may also support decision-making for health systems, including targeted outreach, misinformation monitoring, and fast evaluation of intervention uptake.

Furthermore, the Yang and Bohnet-Joschko [1] treatment of HDBSCAN’s unassigned noise (cluster 1) is a very reasonable analytical choice. Rather than dismissing this noise, they identify it as a candidate “incubator pool.” I believe that embracing these topics can help researchers who want to predict the next wave of sociopsychological determinants before they become new established literature.

## Limitations and Future Work

Despite its advantages over legacy systems, this pipeline introduces some challenges. The transition from graphical user interface–based tools to code-based learning models introduces a computational barrier to entry. There is a risk that evidence synthesis becomes gated behind advanced data science skills. Furthermore, while Yang and Bohnet-Joschko’s [1] dual-level validation ensures semantic coherence, the study lacks an empirical comparison against older baselines (such as LDA or Louvain clustering) on the identical dataset, which would be necessary to quantify the reduction in fragmentation. Additionally, future validation across multiple bibliographic databases (eg, PubMed, Scopus, and Web of Science) would help determine the stability of the pipeline.

Researchers must be careful not to treat these new machine learning algorithms as new inherently opaque systems. While HDBSCAN eliminates the need to guess cluster counts, it introduces hypersensitivity to the minimum cluster size and the minimum sample size. Similarly, UMAP is dependent on neighbor and distance parameters. Future researchers who adopt this methodology will need to optimize and report their parameter selections to ensure their underlying models match their specific discipline. As a researcher and instructor, I would like to stress that addressing this requires shifts in health informatics education, promoting learning that relies on algorithmic logic and mechanics, and not just software operation.

## Conclusion

Yang and Bohnet-Joschko’s [1] study moved successfully from syntactic keyword-matching to semantic vector representation, in an attempt to avoid the limitations of traditional bibliometrics. More importantly, they provided a transparent, reproducible blueprint for future studies. As the volume of medical literature and patient-reported data continues to increase, adopting machine learning pipelines is an imperative for any research designed to extract evidence-driven insights to guide patient care and public health policy.

## Abbreviations

HDBSCAN: hierarchical density-based spatial clustering of applications with noise
LDA: latent Dirichlet allocation
SMM: social media mining
UMAP: uniform manifold approximation and projection
